# Comparative analysis of IscM and IscQu in feline oral squamous cell carcinoma treatment: cytotoxic and apoptotic insights

**DOI:** 10.3389/fvets.2025.1549550

**Published:** 2025-06-12

**Authors:** Huseyin Cakiroglu, Asuman Deveci Ozkan, Gulay Erman, Mehmet Fatih Bozkurt, Sevinc Yanar, Elif Kale Bakir, Yonca Yuzugullu Karakus

**Affiliations:** ^1^Experimental Medicine Research and Application Centre, Sakarya University, Sakarya, Türkiye; ^2^Department of Medical Biology, Faculty of Medicine, Sakarya University, Sakarya, Türkiye; ^3^Health Services Education Research and Application Centre, Sakarya University, Sakarya, Türkiye; ^4^Department of Medical Biochemistry, Institute of Health Science, Sakarya University, Sakarya, Türkiye; ^5^Department of Pathology, Faculty of Veterinary Medicine, Afyon Kocatepe University, Afyon, Türkiye; ^6^Department of Histology and Embryology, Faculty of Medicine, Sakarya University, Sakarya, Türkiye; ^7^Department of Biology, Institute of Science, Kocaeli University, Kocaeli, Türkiye; ^8^Department of Biology, Faculty of Science and Art, Kocaeli University, Kocaeli, Türkiye

**Keywords:** iscador, *Viscum album*, apoptosis, anticancer effect, feline squamous cell carcinoma

## Abstract

**Backround:**

Feline oral squamous cell carcinoma (FOSCC) is the most common malignant oral tumor in cats, characterized by invasive and aggressive behavior regardless of its location. Conventional treatments, including surgery, radiation therapy, and chemotherapy, often yield unsatisfactory outcomes, with tumor progression and tissue destruction frequently leading to euthanasia. In anthroposophical medicine, extracts of *Viscum album* have been developed as complementary cancer treatments, with Iscador, the oldest and most widely used oncological drug, showing promising anticancer potential. This study investigated, for the first time, the cytotoxic and apoptotic effects of IscM and IscQu, two *Viscum album* extracts, on FOSCC cells.

**Methods:**

Using primary cultures of three FOSCC cell lines, cell viability assays were performed to assess cytotoxicity, and the effects on apoptotic cell death, cell cycle arrest, and cellular and nuclear morphology were evaluated. Additionally, mRNA expression levels of *Cyclin D, Cdk4, Bcl-2, Bax,* and *p53* were analyzed.

**Results:**

The results revealed that both IscM and IscQu induced apoptotic cell death and promoted cell cycle arrest in all three FOSCC cell lines tested. IscQu exhibited relatively stronger pro-apoptotic effects compared to IscM, although no significant differences were observed among the cell lines.

**Conclusion:**

These findings suggest that *Viscum album* extracts, particularly IscQu, may exert anti-tumor effects on feline oral squamous cell carcinoma cells *in vitro*.

## 1 Introduction

Squamous cell carcinomas (SCCs) are malignant neoplasms arising from the squamous epithelium (1). They represent one of the main skin tumors and the most common oral neoplasms in both humans and cats (2–5). In both species, SCCs exhibit locally invasive behavior and show higher metastatic rates for oral tumors compared to cutaneous tumors (6–8). Feline skin SCCs (auricle, ears, and nasal planum) are mainly associated with chronic sun exposure (UV radiation), especially ultraviolet B radiation (9). In contrast, SCCs in UV-protected skin and oral tumors may be associated with Felis domesticus papillomavirus-2 and increased protein p16, which are important in cell cycle control (10). Some studies suggest other risk factors for oral SCC (OSCC), such as environmental tobacco smoke, flea collars, and use of pet food containing chemical additives (11, 12). The invasive behavior of OSCC necessitates the adoption of appropriate local treatments. For these reasons, several antitumor techniques are applied in the treatment of cutaneous OSCC, including radiation therapy, cryosurgery, photodynamic therapy, and electrochemotherapy (6, 9, 13, 14). Multimodal approaches associated with surgery have a higher chance of success for oral OSCC (15). However, these tumors are often inoperable at the time of diagnosis and function may be compromised in some cats following radical surgical treatment (5). Studies describe local therapies, including radiotherapy and toceranib, dos anjoswith variable response rates (RR), often short response times, and poor overall survival (5).

Mistletoe (*Viscum album* L.) from the *Santalaceae* family is an evergreen, perennial, hemiparasitic plant that sucks water and nutrients from the host tree. The importance of *V. album* is that it has anticarcinogenic, antidiabetic, antioxidant, blood pressure lowering, sedative, antibacterial, and antiviral, pro-apoptotic, immunomodulatory and cytotoxic effects, and this has been proven by many studies (16–19). In anthroposophical medicine, extracts of *V. album* have been developed to treat cancer patients. The oldest product used is Iscador. Iscador is considered a complementary cancer treatment but is the most widely used oncological drug in Germany. Mistletoe plants growing on different host trees, such as oak for Iscador Qu and apple for Iscador M, determine the type of mistletoe extract. Lectins, viscotoxins, flavonoids, phenolic acids, sterols, lignans, terpenoids, phenylpropanoids, alkaloids and fatty acids are among the active components in mistletoe extract (20). Cytotoxic glycoproteins, mistletoe lectins, are considered the most active components of mistletoe extracts. It has been suggested that the antitumor properties of mistletoe extracts are due to lectins that can stimulate effector cells of the innate and adaptive immune system, such as dendritic cells, macrophages, natural killer cells, as well as B and T lymphocytes (21). In addition, lectins show direct growth inhibition and cell death induction in tumor cells by causing apoptosis or direct necrotic effects (22, 23). Mistletoe has been used as a complementary anticancer therapy in German-speaking countries for over 50 years (20). Nowadays, standardized extracts obtained from the white mistletoe plant are the most promising as an adjunctive drug therapy and are used among patients with various types of cancer. The complementarity of the treatment can be applied as an adjuvant before, during or after chemotherapy. For example, mistletoe extract treatment against human breast cancer is recommended due to its minimal side effects (24). The main compounds with anticancer activity isolated from *Viscum* species are lectins and viscotoxins (25). Despite promising results presented in various clinical trials and biological studies, little is known about the exact mechanism of action of mistletoe extracts on cancer cells (26, 27). One of the studies conducted is that *V. album* extracts have a great potential to sensitize cancer cells to apoptotic cell death for the MCF-7 cell line, which is a human breast cancer cell line (28). However, there is no study showing the effect of *V. album* extracts (Iscador Qu and Iscador M) on feline oral squamous cell carcinoma cells (FOSCC). In this study, it was aimed to evaluate, for the first time, the cytotoxic and apoptotic activities of *V. album* extracts (Iscador Qu and Iscador M) in FOSCC cells, a challenging condition to treat cats.

## 2 Materials and methods

### 2.1 Ethical approval and FOSCC tissue samples

The cats were not used in an experiment; they were operated on to treat cancer. The study made use of tissue samples that had been surgically removed. The Ministry of Environment and Fobasery prepared the “Regulation on Working Procedures and Principles of Animal Experimental Ethics Committees,” which was published in the Official Gazette in February 2014 and had the number 28914. Additionally, the study was explained to cat owners, who then agreed to let their cats participate.

The material of the study consists of three (3) tumor tissues with histopathological and Immunofluorescence (IF) findings of OSCC obtained from 3 cats. The 3-case information is that; Case 1: crossbreed, age 4, sterilized, 3.5 kg bodyweight lesions started from the upper lip trough the philtrum, Case 2: crossbreed, unknown age, sterilized, 3 kg lesions started from the nasal plan and Case 3: crossbreed, age 9, sterilized, 3.8 kg bodyweight lesions started from the upper lip. Tissues taken for oncogram from cats previously diagnosed with OSCC at VetiPati Veterinary Clinic (Sakarya, Türkiye) and VetRoyal Veterinary Clinic (Sakarya, Türkiye) were placed in DMEM medium containing 1% penicillin/streptomycin and stored until the cell culture study began.

### 2.2 Histopathological examination

For hematoxylin–eosin (H&E) staining the tissues were fixed in 10% neutral buffered formalin solution. Fixed tissues were processed routinely and blocked in paraffin. Then, 4–5 micron thick sections were taken from the samples in paraffin blocks with a microtome to normal and silane-coated adhesive slides. Sections were stained with H&E and examined under light microscope. All tumor samples were histopathologically confirmed as squamous cell carcinoma veterinary pathologist prior to primary culture procedures.

### 2.3 Primary cell culture and immunophenotyping of FOSCC cells

Tissue samples stored for cell culture were cut thinly and kept in trypsin/collagenase at 37°C for 20 min with shaking, pipetted vigorously and passed through a 70 μm filter to obtain more individual cells. The cells obtained were grown in DMEM medium by adding 10% Fetal Bovine Serum, 1% Penicillin–Streptomycin and 2 mM glutamine. The cells were incubated in an incubator at 37°C, 95% humidity and 5% CO_2_ until the growth vessels were filled by approximately 80–90%, and the cells were followed under an inverted microscope. The specimens were routinely prepared and embedded in paraffin blocks for histopathological analysis. For light microscopy, tissue slices were cut and stained with hematoxylin and eosin.

Expression of Ki-67 and epidermal growth factor receptor (EGFR), known markers for FOSCC characterization, were used to confirm the neoplastic origin and proliferative activity of the cultured cells (29, 30). The Immunofluorescence (IF) method was used to determine the expression of Ki-67 and EGFR (Supplementary Table 1). For three FOSCC cells obtained by primary cell culture, 5×10^6^ cells were seeded in each well of 6-well plates for each cell group (FOSCC-1, FOSCC-2 and FOSCC-3) and then the cells were incubated in an incubator at 37°C and 5% CO_2_ for 24 h. After incubation, the cells were fixed with 4% paraformaldehyde and treated with permeabilization buffer for 1 h. Then, they were washed twice with Phosphate buffer saline (PBS). After washing, the cells were incubated with anti-ki67 and anti-EGFR antibodies at +4°C overnight. After incubation, they were washed with PBS twice and incubated with Alexa Fluor 488 secondary antibody for 1 h at room temperature and then visualized with a fluorescence microscope (Olympus).

### 2.4 Determination of cytotoxic effects of IscM and IscQu on FOSCC cells

Three oral squamous cell carcinoma (FOSCC-1, FOSCC-2 and FOSCC-3) cells obtained by primary cell culture were treated with Iscador M (IscM, ISCADOR AG, 2981309) and Iscador Qu (IscM, ISCADOR AG, 2981829). Thus, the inhibitory concentration (IC_50_) and the optimal treatment time were determined for IscM and IscQu on FOSCC cells. For this purpose, cells were seeded in 96-well cell culture plates with an average of 5×10^4^ cells and incubated 24 h. At the end of incubation, the different concentrations of IscM and IscQu 0–1,000 μg/mL, (31) were added to each well and incubated 24 and 48 h. Then, Water Soluble Tetrazolium (WST-1) analysis was conducted to determine the cytotoxic effect. According to the WST kit protocol, 10 μL of WST-1 dye was added to each well and waited for 1–4 h, and measurements were made in the Elisa Reader at a wavelength of 450 nm. The viability of the control cells not treated with the component was accepted as 100%, and the viability rates of the experimental cells were expressed as %.

### 2.5 Determination of the effect of IscM and IscQu on the percentage of apoptotic cells and cell cycle arrest in FOSCC cells

The effect of IscM and IscQu on apoptosis and cell cycle distribution in FOSCC-1, FOSCC-2, and FOSCC-3 cells was assessed by flow cytometry using Annexin V/Propidium Iodide (PI) staining and PI-based DNA content analysis, respectively. For both analyses, 5 × 10^5^ cells were seeded into 6-well plates and incubated for 24 h. After treatment with the IC₅₀ concentrations of IscM or IscQu for 24 h, cells were harvested, centrifuged, and washed twice with cold PBS. For apoptosis analysis, cells were stained using the Annexin V-FITC Apoptosis Detection Kit (Abcam, ab14085), following the manufacturer’s instructions. For cell cycle analysis, cells were fixed in cold 70% ethanol overnight at −20°C. After fixation, they were washed and stained with PI/RNase A solution (Abcam Cell Cycle Assay Kit, ab139418) for 30 min at room temperature. Samples were analyzed using a BD FACSCalibur flow cytometer (BD Biosciences), and at least 10,000 events were collected per sample. Data were analyzed using FlowJo v10 software. Appropriate gating was applied to exclude debris and doublets. Quadrant analysis was used for apoptosis (Annexin V vs. PI), and Dean-Jett-Fox model was applied for cell cycle phase distribution.

### 2.6 Determination of the effect of IscM and IscQu on the cell and nucleus morphology in FOSCC cells

The effect of IscM and IscQu on the cell and nucleus morphology of FOSCC-1, FOSCC-2 and FOSCC-3 cells were determined by Acridie Orange (AO) and 4′,6-diamidino-2-fenilindol (DAPI) staining. For this purpose, 2×10^5^ cells were seeded in 6-well plates and the cells were incubated 24 h. Then, IC_50_ concentration of IscM and IscQu were treated to the cells and incubated for 24 h. After incubation the cells were fixed with 4% paraformaldehyde and then washed twice with PBS. Afterwards, cells were stained with AO and DAPI for 30 min and then washed with PBS twice again. They were then visualized with a fluorescence microscope (Olympus).

### 2.7 Determination of the effect of IscM and IscQu on the mRNA expression level in FOSCC cells

The effect of IscM and IscQu on the mRNA expression level in FOSCC-1, FOSCC-2 and FOSCC-3 cells were determined by Real-Time Polymerase Chain Reaction (RT-PCR). For this purpose, 2×10^6^ cells were seeded in T_75_ cell culture flasks and the cells were incubated 24 h. Then, IC_50_ concentration of IscM and IscQu were treated to the cells and incubated for 24 h. After incubation, the total RNA isolation from cells was performed with the kit (Thermo Fisher Scientific) according to the appropriate procedure. The obtained total RNAs were determined by measuring at 260 nm wavelength in a spectrophotometer. cDNA synthesis performed with the “cDNA Reverse Transcription” kit (Thermo Fisher Scientific) by following the manufacturer’s protocol. The obtained cDNA was diluted with nuclease-free distilled water and used in RT-PCR processes via RT-PCR device. For this purpose, *Cyclin D, Cdk4/6, Bcl-2, Bax* and *p53* gene primers were designed based on the reference sequence from the NCBI database using Primer3 software (32) and standard primer-design criteria (Supplementary Table 1). Appropriate reaction mixture (Master Mix, cDNA and relevant primer) was prepared according to the properties of the probe (Syber Green). As the last step, the reaction was performed by adjusting the RT-PCR conditions and cycle number. Changes in the expression levels of the genes were determined using data analysis online software (Qiagen).

### 2.8 Statistical analysis

Differences between experimental groups were evaluated using one-way analysis of variance (ANOVA, Post-Hoc Tukey). Data were evaluated using the “Graph Pad Prism v.9” statistics program. *p* values less than 0.05 were considered statistically significant. To evaluate the mRNA expression analysis results a web-based software^1^ was used.

## 3 Results

### 3.1 H&E and IF findings

**Figure 1:**
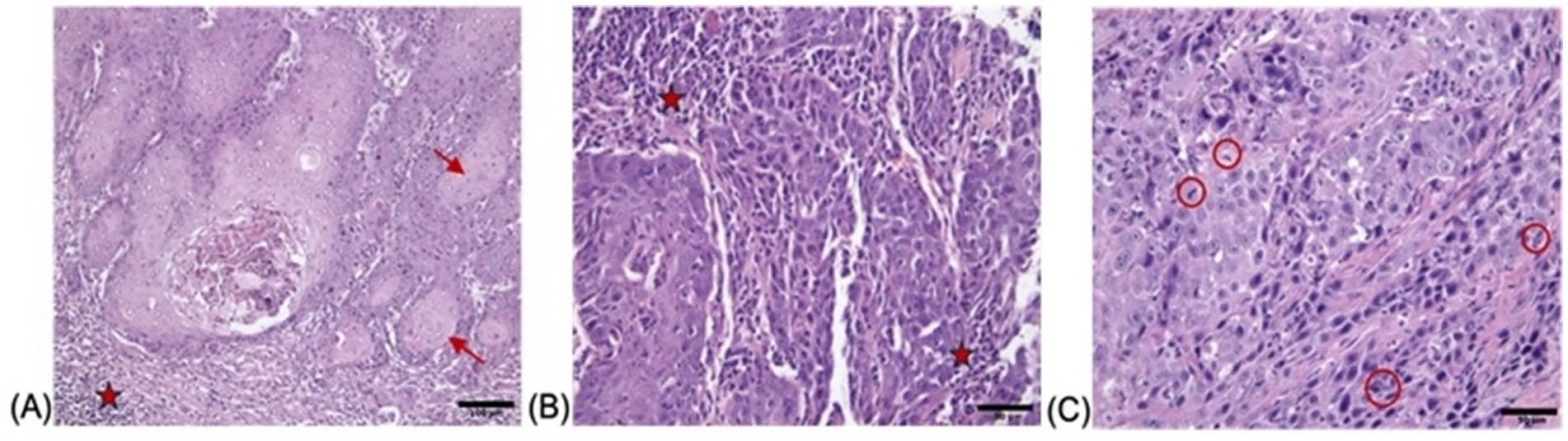
Histopathological findings of FOCC tumor stained with H&E with **(A)** showing nests and islands of neoplastic squamous epithelial cells (→) invading the underlying connective tissue and keratin pearls are indicated by red arrows (×200; scale bar = 100 μm), **(B)** demonstrating sheets of pleomorphic tumor cells (★) with enlarged hyperchromatic nuclei and prominent nucleoli, separated by desmoplastic stroma (×400; scale bar = 50 μm) and **(C)** highlighting numerous mitotic figures (red circles) and apoptotic bodies within the malignant epithelium (×400; scale bar = 50 μm).

H&E staining of the feline oral squamous cell carcinoma tissues revealed distinct histopathological features consistent with malignant squamous cell carcinoma (Figure 1). The first tumor exhibited cellular pleomorphism, with neoplastic cells demonstrating irregular nuclear membranes, prominent nucleoli, and eosinophilic cytoplasm, characteristic of their epithelial origin. Neoplastic nests were observed within the tumor mass, some of which exhibited central keratin pearl formation, further supporting the diagnosis. A mixed inflammatory infiltrate was observed within the stroma adjacent to the tumor nests, indicating a host immune response (Figure 1A). Staining of the second FOSCC case revealed groups of neoplastic squamous cells with moderate pleomorphism, characterized by hyperchromatic nuclei and irregular nuclear-to-cytoplasmic ratios. Inflammatory areas consisted of a mixed infiltrate of neutrophils, plasma cells, and lymphocytes, indicating both acute and chronic immune responses (Figure 1B). In the third FOSCC case, neoplastic squamous cells displayed marked pleomorphism with hyperchromatic nuclei, prominent nucleoli, and frequent mitotic figures. Areas of necrosis were evident, surrounded by a dense inflammatory infiltrate composed of lymphocytes and plasma cells (Figure 1C). These findings were consistent with a well-differentiated squamous cell carcinoma, highlighting the aggressive and invasive nature of this malignancy in feline oral tissues.

**Figure 2:**
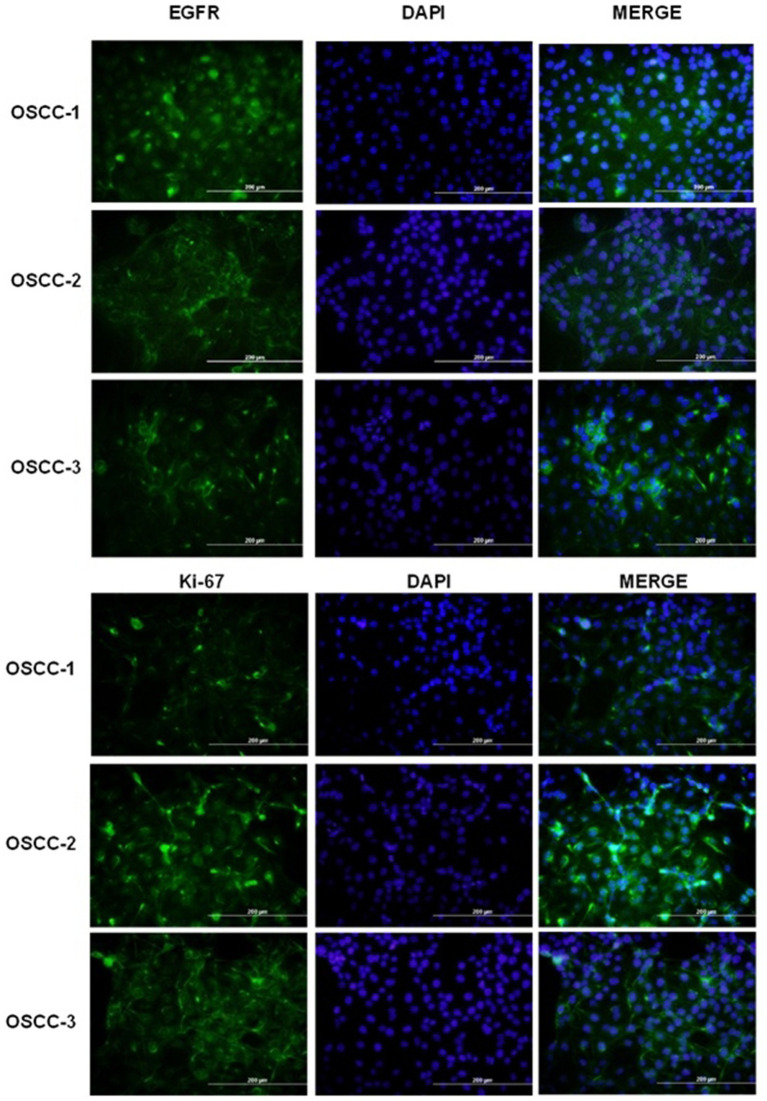
Immunofluorescence analysis of EGFR and Ki-67 expression in primary feline oral squamous cell carcinoma (OSCC) cell lines. Top panels: Representative fields of OSCC-1, OSCC-2 and OSCC-3 cells stained for EGFR (green; left column), counterstained with DAPI to visualize nuclei (blue; middle column), and merged images (right column). Bottom panels: The same cell lines stained for the proliferation marker Ki-67 (green; left column), DAPI (blue; middle column), and merged (right column). All images were acquired at the same magnification; scale bars = 200 μm.

Immunofluorescence (IF) staining was utilized to assess the expression levels and cellular localization of two key markers: epidermal growth factor receptor (EGFR) and Ki-67, within the primary cultured cells (Figure 2). The strong expression of EGFR and Ki-67 confirmed that the cultured cells exhibited molecular features characteristic of OSCC. These results align with previous reports highlighting the diagnostic value of EGFR and Ki-67 in distinguishing OSCC cells from non-malignant counterparts (29, 30). The representative IF images, demonstrating the expression and localization of EGFR and Ki-67 in the isolated primary cultures (Figure 2). Taken together, these findings provide compelling evidence that the established cell cultures are consistent with feline oral squamous cell carcinoma (FOSCC) phenotypes and validate the use of these cells for downstream analyses.

### 3.2 IscM and IscQu decrease the viability of FOSCC cells

**Figure 3:**
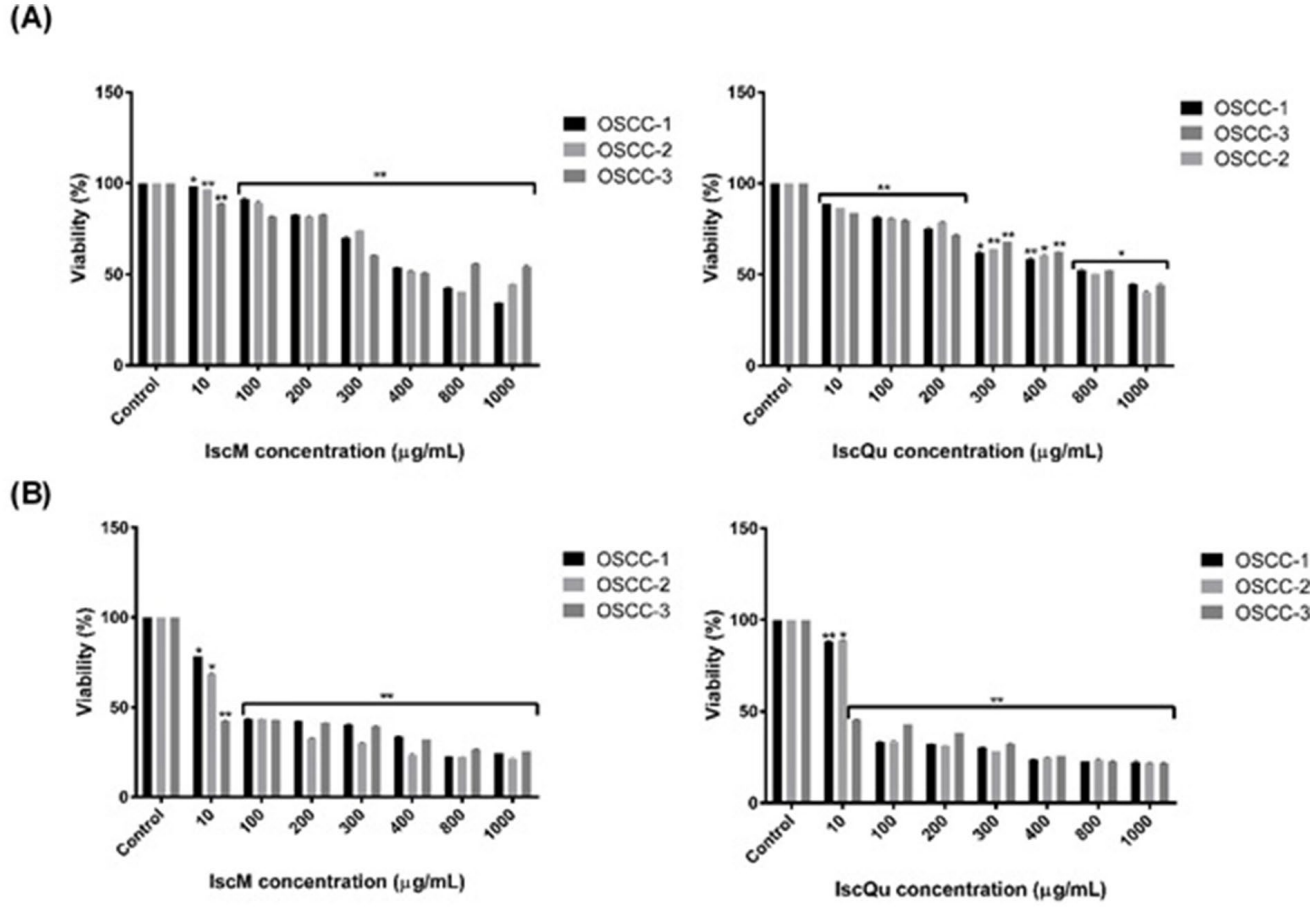
Dose-dependent cytotoxic effects of IscM and IscQu on primary feline OSCC cell viability. **(A)** OSCC-1 (black bars), OSCC-2 (light gray bars) and OSCC-3 (dark gray bars) cells were treated for 24 h with increasing concentrations (10–1,000 μg/mL) of IscM (left) or IscQu (right), and viability was measured by MTT assay and **(B)** The same cell lines were treated for 24 h with the indicated concentrations of IscM (left) or IscQu (right). Data are expressed as percentage of viable cells relative to untreated control. Bars represent mean ± SD of three independent experiments (*p < 0.05, **p < 0.01).

The cytotoxic effects of IscM and IscQu on FOSCC cells were quantitatively assessed using the WST-1 cell viability assay following 24-h and 48-h treatments at various concentrations. As shown in Figure 3, treatment with 800 μg/mL of IscM for 24 h resulted in a significant reduction in cell viability, with viability rates measured at 53.23% ± 0.03 for OSCC-1 cells, 52.98% ± 0.06 for OSCC-2 cells, and 50.12% ± 0.12 for OSCC-3 cells. Similarly, treatment with 800 μg/mL of IscQu yielded comparable cytotoxic effects, with cell viabilities of 52.87% ± 0.14, 49.87% ± 0.09, and 51.24% ± 0.04 for OSCC-1, OSCC-2, and OSCC-3 cells, respectively. To further evaluate the potency of both compounds, the half-maximal inhibitory concentration (IC_50_) values were calculated. For IscM, the IC_50_ values were found to be 323.73 μg/mL, 310.27 μg/mL, and 264.32 μg/mL for OSCC-1, OSCC-2, and OSCC-3 cells, respectively. In comparison, IscQu demonstrated IC_50_ values of 286.78 μg/mL, 461.76 μg/mL, and 438.60 μg/mL for the same cell lines. These values indicate a dose-dependent cytotoxic response, with IscM generally exhibiting a slightly stronger inhibitory effect on cell viability compared to IscQu, particularly in OSCC-3 cells. Furthermore, the 48-h treatments with both compounds resulted in a more pronounced decrease in cell viability across all cell lines, suggesting enhanced cytotoxicity with prolonged exposure. However, based on the balance between effective cytotoxicity and maintaining sufficient cell viability for downstream applications, a 24-h incubation period was selected as the optimal time point for subsequent analyses (Figure 3).

### 3.3 IscM and IscQu increase the percentage of apoptotic cells and cell cycle arrest in FOSCC cells

**Figure 4:**
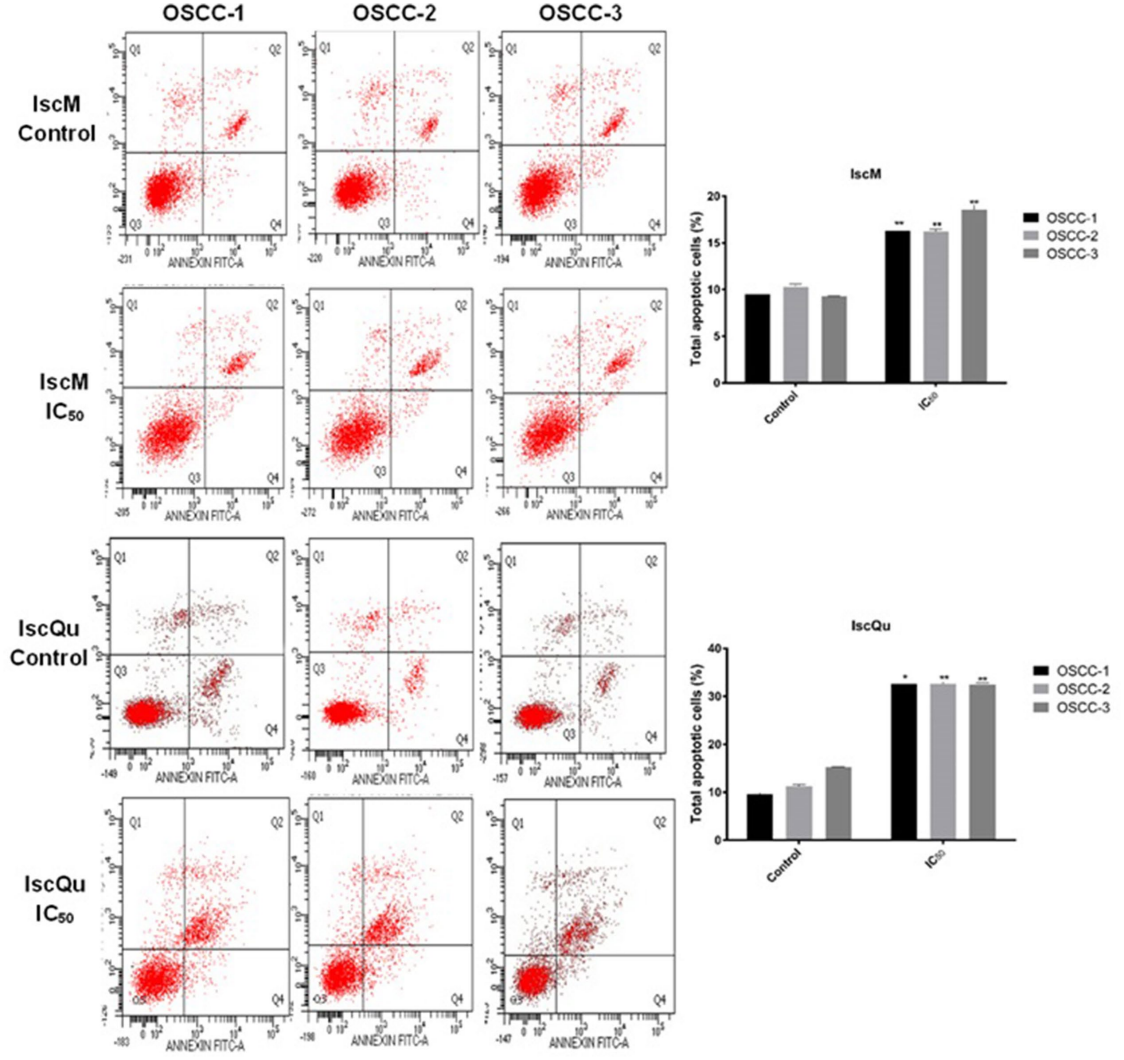
Effect of IscM and IscQu on apoptosis in feline OSCC cell lines. Flow cytometric analysis of apoptosis was performed using Annexin V-FITC/PI staining in three feline OSCC cell lines (OSCC-1, OSCC-2, and OSCC-3) following treatment with either IscM or IscQu at their respective IC₅₀ concentrations for 24 h. Representative dot plots are shown for control (untreated) and treated cells. The lower left (Q3) quadrant indicates viable cells (Annexin V^−^/PI^−^), lower right (Q4) represents early apoptotic cells (Annexin V^+^/PI^−^), upper right (Q2) late apoptotic/necrotic cells (Annexin V^+^/PI^+^), and upper left (Q1) necrotic cells (Annexin V^−^/PI^+^). Bar graphs on the right summarize the percentage of total apoptotic cells (early + late apoptosis) in each group. Data are expressed as mean ± SD from three independent experiments. Bars represent mean ± SD of three independent experiments (*p < 0.05, **p < 0.01).

**Figure 5:**
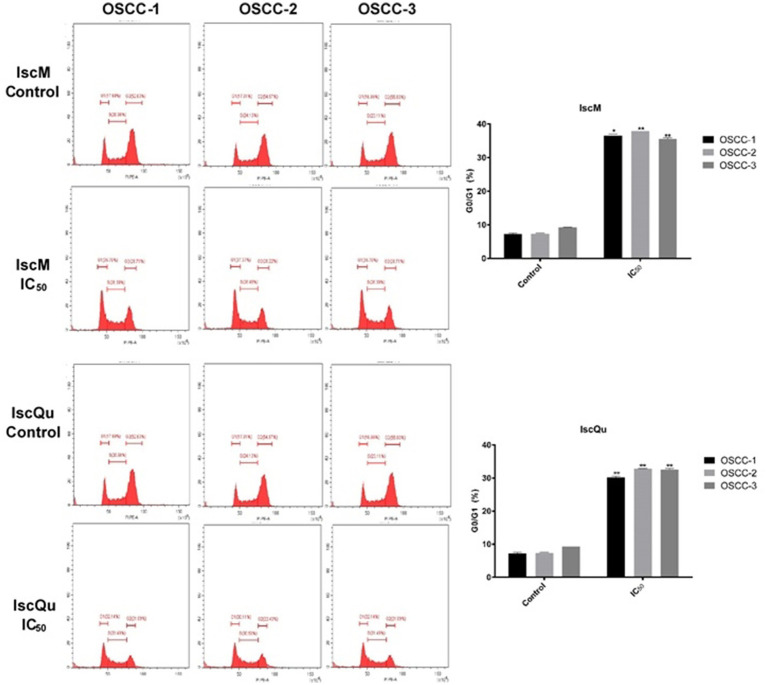
Effects of IscM and IscQu on cell cycle distribution in feline OSCC cell lines. Cell cycle analysis was performed by flow cytometry using propidium iodide (PI) staining in three feline OSCC cell lines (OSCC-1, OSCC-2, and OSCC-3) treated with IscM or IscQu at their respective IC₅₀ concentrations for 24 h. Representative histograms show the distribution of cells in G0/G1, S, and G2/M phases. Bar graphs on the right summarize the percentage of cells in G0/G1 phase. Bars represent mean ± SD of three independent experiments (*p < 0.05, **p < 0.01).

To investigate the impact of IscM and IscQu on apoptosis and cell cycle distribution in FOSCC cells, Annexin V-FITC/PI double staining and flow cytometric cell cycle analyses were performed (Figures 4, 5). The apoptotic response following treatment with IscM revealed a moderate increase in total apoptotic cell population across all three FOSCC cell lines. Specifically, the total apoptotic rates 236 (early + late apoptosis) were found to be 15.93% ± 0.32, 16.86% ± 0.21, and 18.63% ± 0.15 for 237 OSCC-1, OSCC-2, and OSCC-3 cells, respectively, in contrast to the untreated control groups, which 238 exhibited lower baseline apoptotic rates of 9.52% ± 0.23, 10.32% ± 0.029, and 9.35% ± 0.10 (Figure 4). In comparison, IscQu treatment induced a more substantial apoptotic response, with total 240 apoptotic cell rates rising to 32.96% ± 0.12, 34.22% ± 0.19, and 32.43% ± 0.24 in OSCC-1, OSCC-2, 241 and OSCC-3 cells, respectively. These increases were statistically significant when compared to the 242 corresponding control groups (9.56% ± 0.8, 11.8% ± 0.06, and 18.33% ± 0.09), indicating a more potent pro-apoptotic effect of IscQu in FOSCC cells (Figure 4).

Parallel cell cycle analysis revealed that both compounds induced G0/G1 phase arrest, albeit with differing efficiencies. Following IscM treatment, G0/G1 cell population percentages markedly increased to 36.85% ± 0.18, 36.23% ± 0.13, and 37.76% ± 0.10 in OSCC-1, OSCC-2, and OSCC-3247 cells, respectively, compared to their untreated controls, which remained at 7.05% ± 0.13, 7.45% ± 248 0.07, and 7.48% ± 0.16 (Figure 5). IscQu treatment also promoted G0/G1 arrest, although to a 249 slightly lesser extent, with rates of 30.05% ± 0.12, 32.79% ± 0.15, and 32.76% ± 0.18 observed in the 250 respective cell lines.

Statistical analyses confirmed that both IscM and IscQu significantly induced apoptotic cell death and G0/G1 phase cell cycle arrest in FOSCC cells. Notably, IscQu was more effective in triggering apoptosis, while IscM demonstrated a greater ability to arrest the cell cycle in the G0/G1 phase.

### 3.4 IscM and IscQu change the cell and nucleus morphology in FOSCC cells

**Figure 6:**
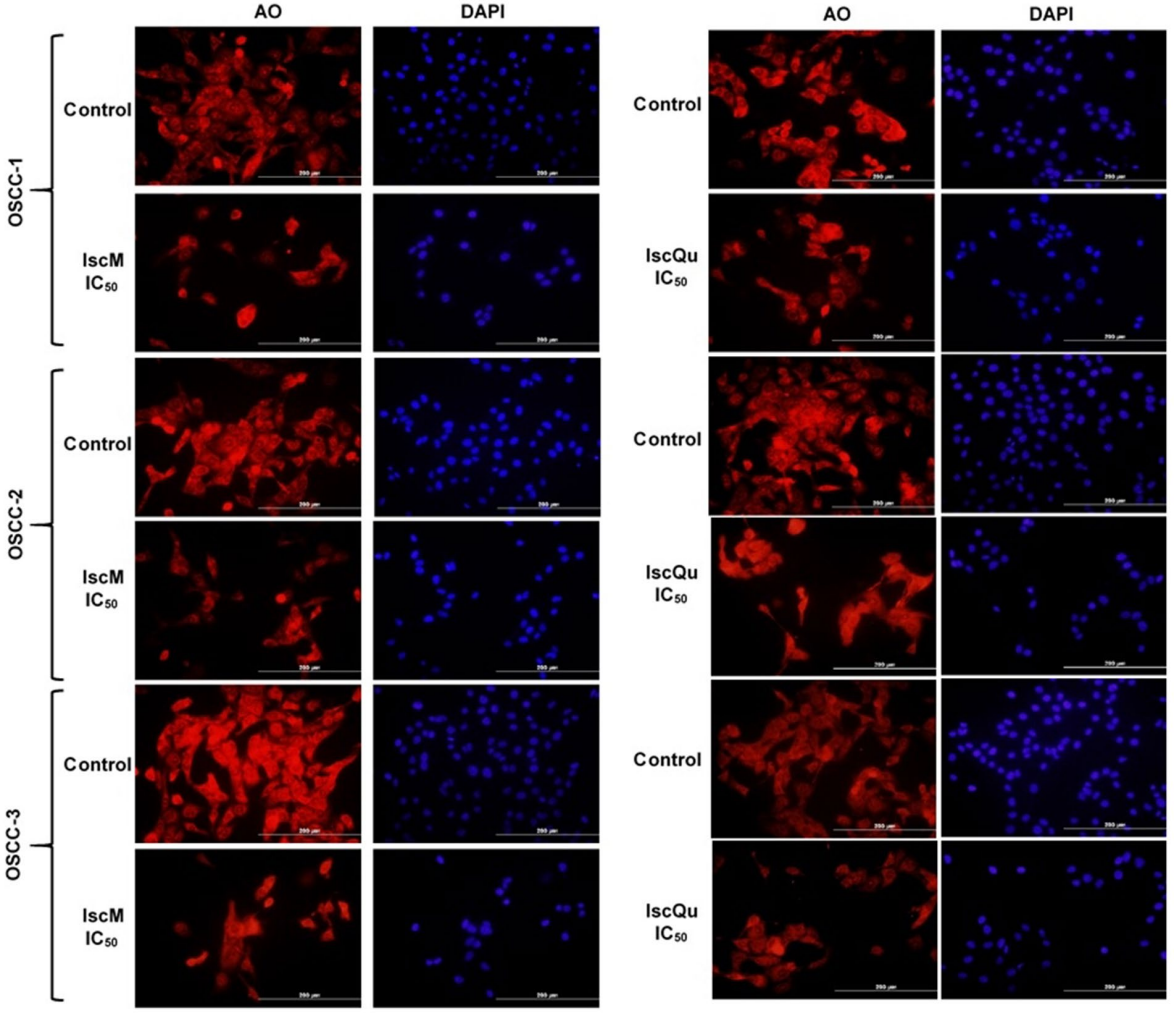
Fluorescence microscopy analysis of nuclear and cytoplasmic morphological changes in feline OSCC cells treated with IscM and IscQu. Feline OSCC-1, OSCC-2, and OSCC-3 cells were treated with IC₅₀ concentrations of IscM or IscQu for 24 h. Following treatment, cells were stained with acridine orange (AO; red) to visualize cytoplasmic morphology and DAPI (blue) to detect nuclear condensation and fragmentation. Scale bars = 200 μm.

To further evaluate the morphological changes associated with apoptosis induced by IscM and IscQu in FOSCC cells, acridine orange (AO) and 4′,6-diamidino-2-phenylindole (DAPI) staining were performed, enabling the visualization of both cytoplasmic and nuclear alterations (Figure 6). Following treatment with both compounds, marked morphological disruptions were observed when compared to the untreated control group. Specifically, treated cells exhibited characteristic features of apoptosis, including membrane blebbing, chromatin condensation, nuclear fragmentation, cytoplasmic shrinkage, and loss of normal cellular architecture. AO staining revealed cytoplasmic shrinkage and membrane budding, while DAPI staining highlighted pronounced nuclear condensation and fragmentation—more prominently in IscQu-treated cells. These morphological hallmarks of apoptosis were consistently observed across all three FOSCC cell lines, further confirming the pro-apoptotic effects of IscM and IscQu. Notably, apoptotic morphological features were more frequent and pronounced in cells treated with IscQu compared to IscM, which aligns with the flow cytometry data indicating a higher apoptotic cell rate in the IscQu group. Despite this difference in extent, both treatments resulted in a comparable pattern of apoptotic morphological changes among the different FOSCC cell lines. In summary, these morphological observations corroborate the findings from the Annexin V-FITC/PI assays and support the conclusion that Iscador compounds, particularly IscQu, promote apoptosis in FOSCC cells through characteristic morphological changes indicative of programmed cell death.

### 3.5 IscM and IscQu increase the apoptosis- and cell cycle-related mRNA expression in FOSCC cells

**Figure 7:**
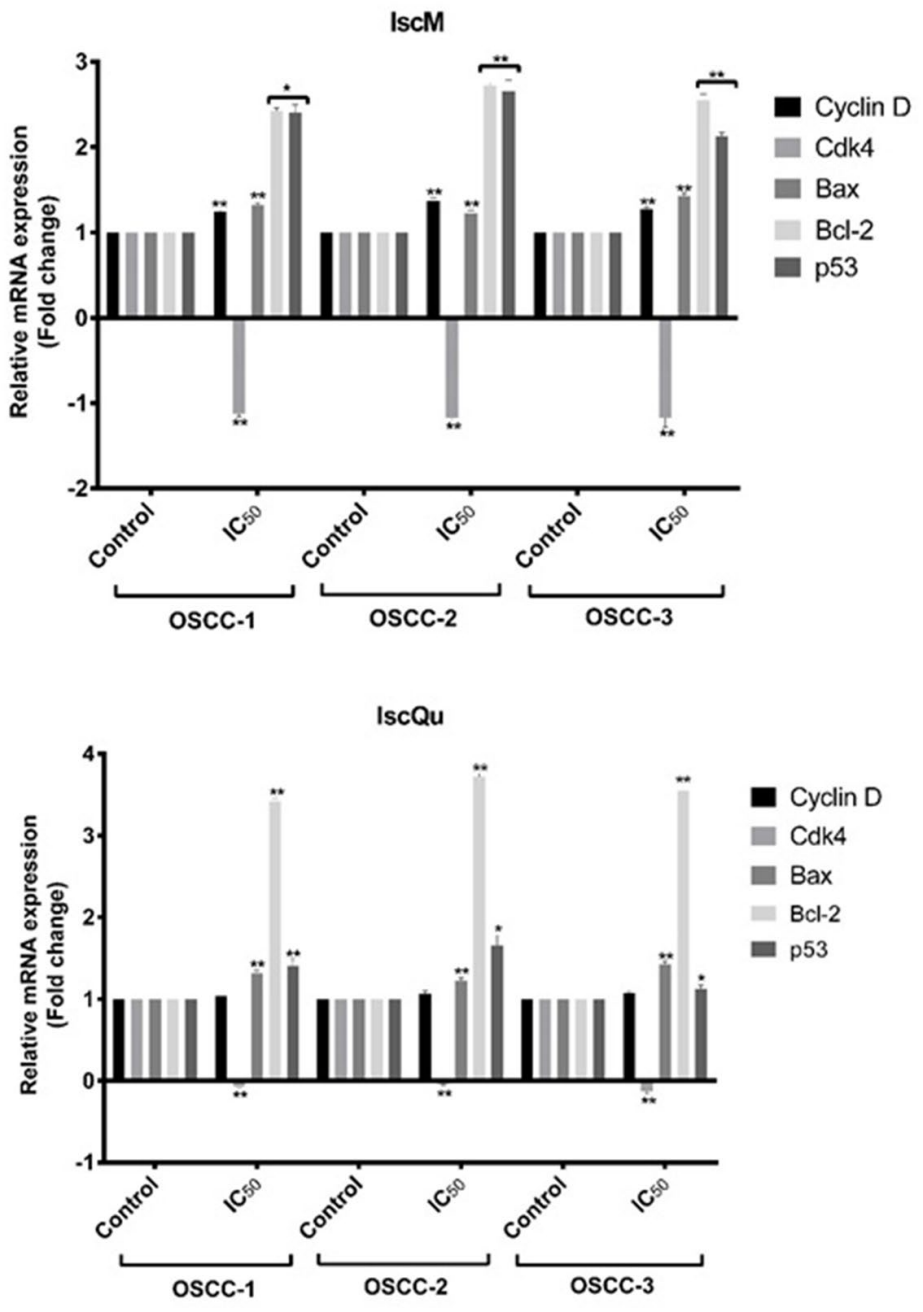
Effects of IscM and IscQu treatments on mRNA expression levels of cell cycle and apoptosis-related genes in OSCC cell lines. Relative mRNA expression levels of *Cyclin D, Cdk4, Bax*, *Bcl-2*, and *p53* were assessed by RT-PCR in three OSCC cell lines (OSCC-1, OSCC-2, OSCC-3) following treatment with IC₅₀ doses of IscM (top panel) and IscQu (bottom panel). Expression levels are presented as fold changes relative to the untreated control group. Bars represent mean ± SD of three independent experiments (*p < 0.05, **p < 0.01).

To investigate the molecular mechanisms underlying the apoptotic and cell cycle effects of IscM and 276 IscQu in FOSCC cells, the mRNA expression levels of key regulatory genes (*Cyclin D, Cdk4, Bcl-2,* 277 *Bax,* and *p53*) were evaluated using reverse transcription polymerase chain reaction (RT-PCR) 278 analysis (Figure 7). The analysis revealed that *Cdk4* mRNA expression, a gene critical for G1 to S 279 phase transition in the cell cycle, was notably downregulated in response to both IscM and IscQu 280 treatments (−1.3- and −0.3- fold respectively) across all FOSCC cell lines. In contrast, *Cyclin D* 281 mRNA expression remained largely unchanged, maintaining levels comparable to untreated controls. 282 Furthermore, a significant upregulation of *p53* mRNA expression was detected following both 283 treatments (2.2- and 3.7- fold, respectively). In terms of apoptosis-related genes, an unexpected trend 284 was observed: *Bax*, a pro-apoptotic gene, was slightly downregulated (1.1-fold and 1.2- fold, 285 respectively), while *Bcl-2*, an anti-apoptotic gene, was upregulated (2.5- and 3.5- fold, respectively) 286 in response to both IscM and IscQu. RT-PCR results demonstrated that both IscM and IscQu 287 modulate the expression of genes involved in cell cycle control and apoptosis. The upregulation of 288 *p53* and downregulation of *Cdk4* are consistent with the observed G0/G1 cell cycle arrest, while the 289 discrepancies in *Bax* and *Bcl-2* expression highlight the complexity of apoptosis regulation at the 290 gene level. These molecular changes further support the flow cytometric and morphological findings, 291 confirming that Iscador treatments influence FOSCC cell fate through coordinated gene expression 292 alterations.

## 4 Discussion

Squamous cell carcinoma is the most common malignant tumor of the oral cavity in cats, accounting for 60–70% of all oral malignancies (5). Oral squamous cell carcinoma (OSCC) in cats most commonly involves the lingual region and the toothed jaws and may present as a necrotic ulcerative lesion or a hard nodular swelling, usually associated with high local invasiveness and early bone lysis (5). Although regional and distant metastases have been reported, death most often results from complications related to the primary tumor before metastatic disease has had the opportunity to become clinically significant (33). Diagnosis is often late due to the location and rapid tumor progression, greatly limiting the effectiveness of treatments including surgery, radiation therapy, and chemotherapy. The prognosis in most cats is poor and even with a multimodal therapeutic approach, the median survival time rarely exceeds 12 months (34). In this sense, the approach to traditional treatments is important and, in this study, the cytotoxic and apoptotic effects of Iscador, the oldest product of *V. album* extracts, which has the potential to be an alternative treatment, were demonstrated for the first time. The cytotoxic, apoptotic, and cell cycle-modulating effects of Iscador M (IscM) and Iscador Qu (IscQu) extracts on feline oral squamous cell carcinoma (FOSCC) cells were comprehensively evaluated through molecular, morphological, and functional assays. The results demonstrated that both extracts exerted significant anti-tumor effects, with notable differences in their mechanisms of action and potency.

The WST-1 viability assay revealed a dose-dependent cytotoxic effect of both IscM and IscQu, with slightly lower IC50 values observed in IscM-treated cells. These findings suggest that both mistletoe extracts impair cellular proliferation in FOSCC cells, which is consistent with previous studies highlighting the anti-proliferative effects of *Viscum album* preparations in various cancer cell types (23–25). The greater cytotoxic effect observed with prolonged exposure (48 h) further supports the potential of time-dependent enhancement in therapeutic efficacy.

Recent studies have shown that mistletoe (*Viscum album*) extracts, particularly those derived from oak, possess significant anti-cancer properties through the induction of apoptosis and the modulation of cell cycle progression. Mistletoe lectins, the active compounds in these extracts, have been reported to exhibit potent pro-apoptotic effects in various cancer cell lines. These lectins are known to bind to glycoproteins on the cell membrane, initiating a cascade of signaling events that lead to programmed cell death (35). Furthermore, mistletoe extracts are also known to induce cell cycle arrest by targeting key regulatory proteins involved in cell division, such as cyclins and cyclin-dependent kinases (CDKs) (20). Flow cytometric analysis of apoptosis using Annexin V-FITC/PI staining indicated that IscQu induced a significantly higher level of apoptotic cell death than IscM in all three FOSCC cell lines. This finding aligns with earlier reports highlighting the higher lectin content and stronger pro-apoptotic activity of oak-derived mistletoe extracts like IscQu. In contrast, cell cycle analysis revealed that IscM was more effective at inducing G0/G1 cell cycle arrest, suggesting a differential effect on cellular mechanisms regulating proliferation. These findings suggest that while both extracts promote anti-cancer effects, they may do so through complementary biological pathways—one favoring apoptosis, and the other inhibiting cell cycle progression.

The p53 protein, encoded by the p53 onco-suppressor gene, prevents proliferation of genetically damaged cells, thus counteracting oncogenic transformation and tumor growth (36). Under unstressed physiological conditions, the p53 protein has a half-life of 5–20 min in most cell types and is maintained at a low level (37). Mutations in p53 cause conformational changes that stabilize the protein (43). Somatic mutations in the p53 gene are the most common alterations in human head and neck squamous cell carcinoma (HNSCC), detected in up to 85% of cases, and have been associated with tobacco carcinogenesis (38). Similarly, the data obtained from this study showed that the mRNA expression of the cell cycle-related *p53* gene increased, aligning with the literature. This upregulation of *p53* suggests activation of a DNA damage response pathway and supports the cell cycle arrest observed, particularly in the G0/G1 phase after IscM treatment.

Cellular proliferation follows an orderly progression through the cell cycle, regulated by protein complexes composed of cyclins and cyclin-dependent kinases (CDKs) (39). Cyclins are a family of cell cycle control proteins that regulate cell cycle progression by associating with and activating CDKs (40). Since the major regulatory events leading to mammalian cell proliferation and differentiation occur during the transition from G0 to G1 phases or from G1 to S phase during the cell cycle, deregulated expression of G1 or G1/S phase cyclins or their associated CDKs may result in loss of cell cycle control and thus contribute to neoplastic transformation (39, 40). The data obtained from this study also showed that cell cycle arrest occurred especially in G1 in IscM and IscQu treated groups and that there was a decrease in the expression of Cyclin D and Cdk4 mRNA in gene expression analyses. These data show us, consistent with the literature, that Iscador promotes cell cycle arrest and that it does this by preventing or reducing the formation of the Cyclin D/CDK4 complex, which specifically performs cell cycle arrest in G1. This further supports the role of *Viscum album* extracts in inhibiting cell cycle progression and tumor growth by targeting key regulatory proteins in the G1 phase.

At the molecular level, RT-PCR analysis revealed that both IscM and IscQu treatments led to a significant downregulation of *Cdk4*, while *Cyclin D* expression remained unchanged, suggesting that inhibition of G1 phase progression was primarily mediated by suppression of Cdk4. In addition, the expression of *p53* was upregulated in all treatment groups, supporting the activation of DNA damage response pathways and cell cycle arrest. Interestingly, a downregulation of the pro-apoptotic gene *Bax* and upregulation of the anti-apoptotic gene *Bcl-2* were observed, which appeared contradictory to the functional apoptosis data. This discrepancy may indicate the involvement of post-transcriptional regulation, p53-mediated apoptosis pathways independent of the classical Bcl-2/Bax balance, or delayed gene-level responses. Such complexity underscores the multifaceted nature of cellular apoptosis regulation and highlights the necessity of integrating transcriptomic data with phenotypic observations.

Despite the promising findings, several limitations of this study should be acknowledged. All experiments were conducted *in vitro* using a limited number of primary FOSCC cell lines, which may not fully represent the tumor heterogeneity found in clinical cases. Additionally, gene expression was assessed only at the mRNA level, and no protein-level validation (e.g., via Western blot) was performed to confirm the functional activity of the observed gene expression changes. Furthermore, the mechanisms underlying the discrepancies in pro- and anti-apoptotic gene regulation warrant further investigation. Future *in vivo* studies and pathway-specific molecular analyses will be essential to validate and expand upon these findings.

In conclusion, both Iscador M and Iscador Qu demonstrate significant anti-cancer activity against FOSCC cells through complementary mechanisms. The WST-1 viability assay revealed a dose-dependent reduction in cellular proliferation indicating a potent cytotoxic effect that is further enhanced with prolonged treatment. Flow cytometric analyses confirmed that while IscQu induces a higher rate of apoptotic cell death as evidenced by Annexin V-FITC/PI staining, IscM predominantly leads to G0/G1 cell cycle arrest, underscoring a differential modulation of cell cycle regulatory mechanisms. Morphological evaluations provided visual confirmation of apoptotic features. In addition, RT-PCR analyses indicated that both treatments promote cell cycle arrest by downregulating *Cdk4* expression without significantly affecting *Cyclin D* levels, while upregulation of *p53* supports the activation of stress response and DNA damage pathways (41, 42). Although the expression of pro-apoptotic (*Bax*) and anti-apoptotic (*Bcl-2*) genes showed a complex pattern, the overall gene expression data support the functional findings of increased apoptosis and cell cycle inhibition. These findings confirm that Iscador exert their anti-cancer effects on FOSCC cells by disrupting key processes of cellular proliferation and survival. The data suggest that IscQu may have superior pro-apoptotic properties, whereas IscM appears to induce a stronger cell cycle arrest. This complementary action emphasizes the potential utility of these extracts as adjunctive agents in the management of FOSCC.

## Data Availability

The data underlying this study cannot be made publicly available due to confidentiality restrictions. Data are available from the corresponding author upon reasonable request.

## Footnotes

^1^ https://geneglobe.qiagen.com/us/analyze
