# Supplementary material for: Comparative analysis of IscM and IscQu in feline oral squamous cell carcinoma treatment: cytotoxic and apoptotic insights
Source: Front Vet Sci. 2025 Jun 12;12:1549550. doi: 10.3389/fvets.2025.1549550 (PMC12199264; doi:10.3389/fvets.2025.1549550)
Supplement: Supplementary file 1 [file Table_1.DOCX]

Supplementary Material

**Supplementary Table 1.** Primary antibodies used for immunofluorescence (IF) analysis.

| **Antibody** | **Manufacturer** | **Code** | **Dilution** |
| --- | --- | --- | --- |
| EGFR | Elabscience | Monoclonal Antibody (AN005400L) | 1:200 |
| Ki-67 | Elabscience | Polyclonal Antibody (E-AB-31291) | 1:500 |

**Supplementary Table 2.** Primer sequences used for gene expression analysis.

| **Gene** | **Sense primer (5' → 3')** | **Anti-sense primer (5' → 3')** |
| --- | --- | --- |
| **Cyclin D** | AAATGGGCTCCCTCTATCAGTTC | TCTGCTTGGTGGTTTGCTACGAC |
| **Cdk4/6** | CCAGGATGAGGACCCAAGCA | TCCCGACCATTGCTGTTTCC |
| **Bcl-2** | TGAGGTTTATTGGCACCTCC | CAGCTGCACCTGACGCCCTT |
| **Bax** | CCCAGCCTCCGTTATTCTGGA | TGGACGGACAGAATACACCA |
| **p53** | TGGACGGACAGAATACACCA | TGGACACAAAGTGGGACATC |
| **β-actin** | AAGTCCCTCACCCTCCCAAAAG | AAGCAATGCTCACCTTCCC |
